# Topography of Bone Erosions at the Metatarsophalangeal Joints in Rheumatoid Arthritis: Bilateral Mapping by Computed Tomography

**DOI:** 10.7759/cureus.15823

**Published:** 2021-06-22

**Authors:** Paolo Simoni, Sakina Moussaddykine, Olivier Malaise, Selma Ben Mustapha, Maria Pilar Aparisi Gómez, Alessandro De Leucio

**Affiliations:** 1 Radiology, Queen Fabiola Children's University Hospital - Université Libre de Bruxelles, Brussels, BEL; 2 Rheumatology, Centre Hospitalier Universitaire de Liège - Université de Liège, Liège, BEL; 3 Radiation Oncology, Centre Hospitalier Universitaire de Liège - Université de Liège, Liège, BEL; 4 Radiology, Auckland City Hospital, Auckland, NZL; 5 Radiology, Hospital Vithas Nueve De Octubre, Valencia, ESP; 6 Radiology and Medical Imaging, Queen Fabiola Children's University Hospital, Brussels, BEL

**Keywords:** rheumatoid arthritis, erosion, metatarsophalangeal joint, computed tomography, mri

## Abstract

Objectives: To describe the bilateral anatomical location of bone erosions (BE) at the metatarsophalangeal joints in patients with rheumatoid arthritis using computed tomography.

Materials and methods: Eighteen consecutive patients with established rheumatoid arthritis prospectively underwent computed tomography of both forefeet. Each joint surface of the metatarsal heads (MTH) and the proximal phalangeal bases were divided into four quadrants: superior, plantar, tibial, and fibular. The number of BE was cumulatively counted per patient, side, joint, per joint surface, and quadrant. Descriptive statistics, paired and unpaired samples t-tests, Pearson's correlation coefficients, ANOVA 2, and variance component analysis were performed.

Results: There were 288 BE at the MTH and 66 at the proximal phalanges. The number of BE in one forefoot was a poor predictor of the absolute number of BE on the contralateral foot "r=0.54" and was unrelated to symptoms.

The superior quadrants were less frequently affected than other quadrants for both the MTH "p<0.0001" and proximal phalanges "p<0.001." The tibial quadrant showed a higher number of BE compared to all other quadrants for MTH "p<0.03," proximal phalanges "p<0.01, and for the metatarsophalangeal joint as a whole "p<0.0001." Plantar and fibular quadrants were equally affected "p<0.05."

Conclusion: BE were found more frequently on the tibial side of the MTH in patients with rheumatoid arthritis.

## Introduction

Rheumatoid arthritis (RA) is a chronic inflammatory disease causing joint swelling, tenderness, chronic disability, and reduced life expectancy [1]. RA has a worldwide prevalence of 0.5-1.0% [2]. Most RA patients are diagnosed between the age of 35 and 50 years [2]. Bone erosions (BE) play a key role in joint damage, causing intra-articular bone loss and joint failure [3].

The metatarsophalangeal joints (MTPJ) are the most affected anatomical areas of the lower feet [4]. In 57% of patients with established RA, the number of BE at the MTPJ on radiographs is directly related to the degree of walking limitation and the long-term quality of life [5]. As in other joints, in the MTPJ, BE were classically associated with the presence of the synovial pannus penetrating the subchondral bone at the so-called joint "bare areas” [6]. However, the advent of MRI highlighted the importance of the inflammatory edema of the subchondral bone marrow in the pathogenesis of BE [7]. However, as proven in different joints, neither synovial pannus nor subchondral bone edema is spatially related to the specific distribution of the BE [8].

In 1965, Martel et al. [9] first suggested that BE might be more frequent in joint areas exposed to higher mechanical demand. More recently, Siddle et al. [10] demonstrated that BE were statistically more frequently found at the plantar aspect of the metatarsal heads (MTH) on the MRI assessment limited to the more symptomatic forefoot. These findings support the hypothesis that mechanical demand for the BE can determine the BE location at the MTPJ. However, this study was limited to the more symptomatic side.

Computed tomography (CT) enables a fast and low-dose examination of both forefeet in the same patients [11]. In addition, CT, due to its spatial resolution and its exquisite capability to assess the mineral structure of the bone, is considered the gold standard imaging technique to detect BE in clinical settings [11-14].

This study aims to provide a first description of the bilateral anatomical distribution of BE at the MTPJ using computed tomography to gauge the hypothesis that mechanical demand determines their distribution.

## Materials and methods

Study population and image collection

We retrospectively reviewed the CT images of both forefeet of 18 consecutive outpatients referred to the Rheumatology Department of the University Hospital of Liège, Belgium, from March to May 2014. This population was assessed by CT to compare the accuracy of tomosynthesis with that of X-rays, using CT as the gold standard in a previously published prospective study [11]. All patients enrolled met the American College of Rheumatology/European League Against Rheumatism criteria for RA [1]. All subjects were older than 18 years. All patients had established RA, defined as RA with a diagnosis older than six months. To describe the anatomical location of BE of both feet, CT images were retrieved from the local Picture Archiving and Communicating System (PACS). The CT images were reviewed by one musculoskeletal radiologist (PS) with 16 years of experience, blinded to the demographic and clinical data.

The Local Ethics Committee approved this retrospective study, and patients’ written consensus was waived for this retrospective study.

CT technique

CT examinations were performed using a 16-slice multidetector CT (Sensation 16, Siemens, Erlangen, Germany). Patients were positioned supine with ankles in slight extension, the heels resting on the table, and the feet placed on a cushion. Feet were scanned from the ankle joint to the toe tips. Native images were obtained in a "feet first and prone" position, the ankle forming a 90° angle with the legs. A standard protocol using 120 kV, an effective tube current-time product of 108 mAs, a pitch of 0.5, and a collimation of 6 × 0.75 mm^2^ was used. Native slices of 0.75 mm were obtained using the U70 Kernel. The mean dose-length product calculated on population was 206.3 ± 24.2 mGy*cm.

Study population and image collection

All images were anonymised and reviewed using a medical workstation (IMPAX, Agfa HealthCare, version 6.5.3.1509, Mortsel, Belgium) equipped with a pair of 5-megapixel medical screens (MDCC-6230, Barco, Kortrijk, Belgium with a physical size of 654 × 409 mm^2^ and a native resolution of 3280 × 2048 pixels). Multiplanar reconstruction (MPR) images in all required plans were performed.

MPR images were manually obtained by the reviewing radiologist, using the Average Intensity Projection and a slice thickness of 2 mm. The window was set with window level centred at 330 Hounsfield Unit (HU) and window width of 1400 HU.

Operational definition

Bone Erosions

According to the international recommendations [7], BE was defined as a bony defect in the joint or an abrupt loss of the subchondral bone plate on at least two planes on CT images on MPR.

BE Topography

**Figure 1:**
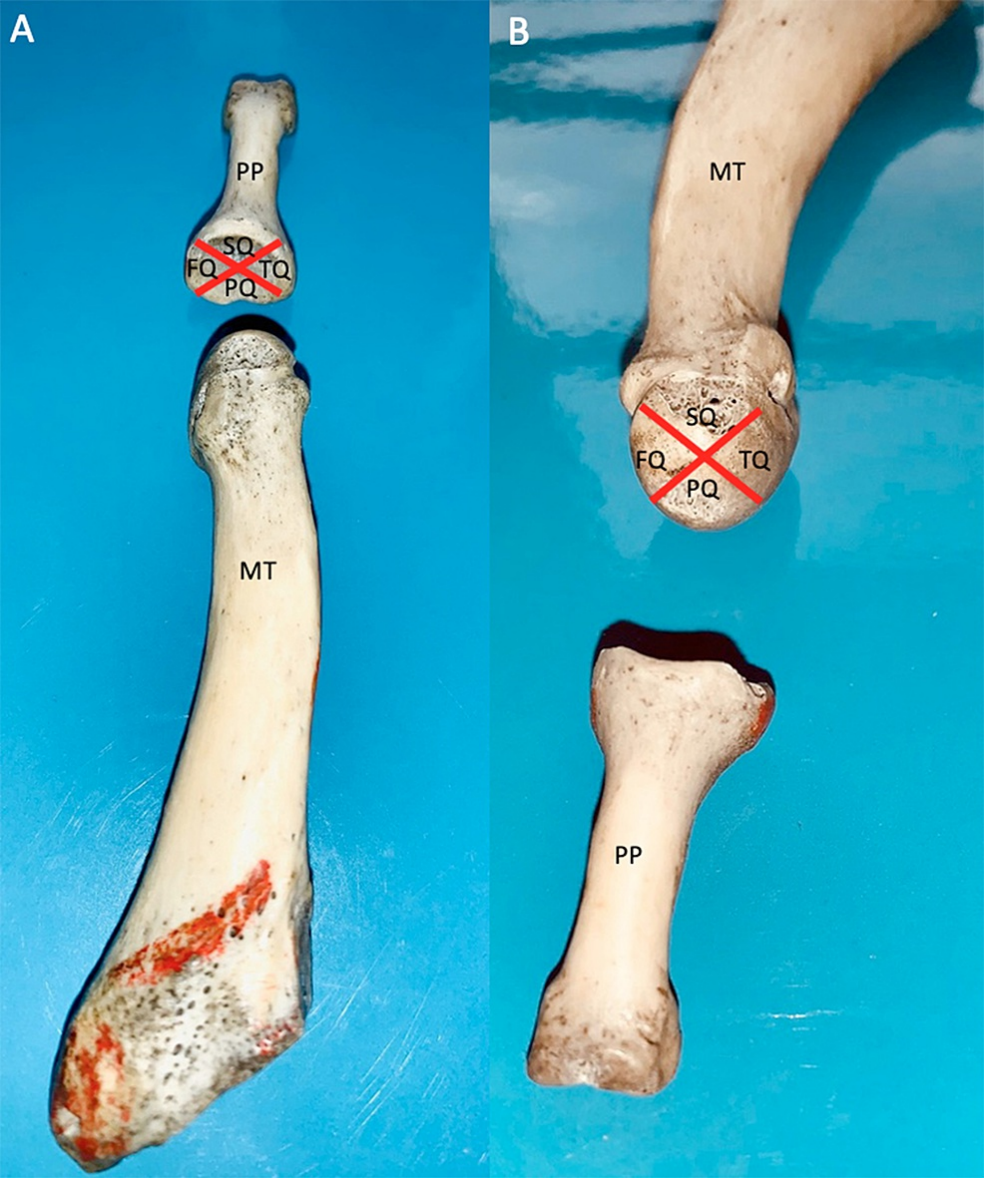
Representation of the quadrants of the metatarsophalangeal joint on the anatomical specimen. (A) Division in quadrants of the proximal phalanx joint surface and (B) division in “pie-slice” quadrants of the metatarsal head joint surface. PP: proximal phalanx, M: metatarsal bone, SQ: superior quadrant; TQ: tibial quadrant (medial), PQ: plantar quadrant, FQ: fibular quadrant (lateral).

**Figure 2:**
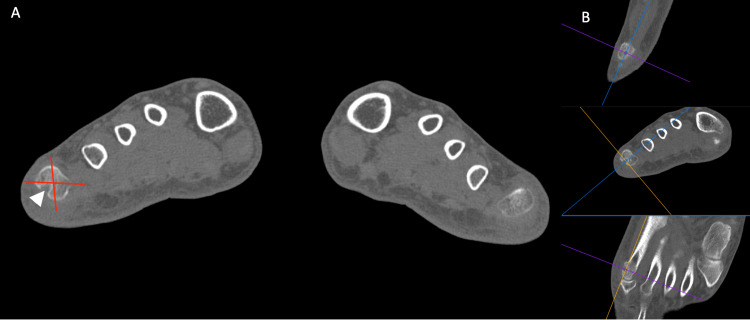
Computed tomography of both forefeet with multi-planar reconstructions. (A) Bone erosion (white arrowhead) located at the fibular quadrant (FQ) of the fifth metatarsal head of the right foot (image acquired at 2 mm of thickness and reconstructed at 2 mm of thickness; (B) the multi-planar reconstruction (MPR) centered on the focal bone loss confirms the presence of the bone erosion. The red lines represent the division in quadrants.

Each joint surface, including the MTH and the bases of the proximal phalanges (PP), was divided into four quadrants, including a superior (dorsal), inferior (plantar), medial (tibial), and lateral (fibular) quadrants, according to previous studies (Figures 1 and 2) [8,15]. This joint mapping allows separating the plantar quadrant of the joint subjected to the weight of the body in standing position from the tibial (i.e., medial) and the fibular (i.e., lateral) quadrants where the mechanical demand is elicited by the entheses of the collateral ligaments [8].

Erosions larger than a quadrant were attributed to the quadrant in which the epicentre of the erosion was located. The epicentre of erosions was defined as the midpoint of the main axis of the erosion in the axial plane (perpendicular to the long axis of the metatarsal). If there was more than one erosion in a quadrant, we considered only the largest one. For both the MTH and PP, the analysis was extended to the first centimetre from the articular surface, as in previous studies [10].

Multi-level Method to Classify BE Topography

To analyze the anatomical distribution (topographical analysis) of BE, each erosion was classified according to the following multi-level method: (i) number of BE per patient; (ii) number of BE per side; (iii) number of BE on MTH; (iv) number of BE on proximal phalanges; (v) number of BE per quadrant.

Statistical analysis

The statistical analysis was carried out by a professional biostatistician (LS) using SAS software (SAS Institute, North Carolina, USA). Descriptive statistics (mean, standard deviation), t-test for paired and unpaired samples, Pearson’s correlation coefficients, two-way analysis of variance (ANOVA 2) to compare the averages of the same sample, and a variance component analysis were carried out. The results were considered significant if p < 0.05.

## Results

Demographics and clinical features

In the population of 18 patients, there were 12 women and 6 men. There was no significant difference in age between men and women (64.2 ± 10.7 vs 61.8 ± 17.0; p=0.71). The mean disease duration was higher in women (13.2 ± 9.1 years) than in men (5.5 ± 4.7 years; p= 0.027). At the clinical examination of the MTFJ, 9 out of 18 patients (50%) were asymptomatic, 2 out of 18 (11.1%) presented pain and/or of their left forefoot, 7 out of 18 (38.9%) presented pain or swelling bilaterally.

Analysis of the Number of BE by the Multilevel Model

Number of BE per patient: In the population of 18 patients, CT revealed 324 erosions at the MTPJ. A total of 258 BE were found at the MTH (80%), while 66 were detected at the base of the proximal phalanx (20%; p>0.0001). The mean number of BE found on CT for each patient at the MTPJ (including both forefeet) was 18±7.8. The number of BE in one forefoot was a poor predictor of the absolute number of BE of the contralateral one (r=0.54) even if the BE of the forefeet was statistically correlated in each patient (p=0.021). At ANOVA 2 analysis was no statistically significant correlation between the number of BE and demographic covariates such as patients’ pain and swelling, age, gender, symptomatic/asymptomatic side(s), and the mean disease duration.

The number of erosions per side: There was no statistically significant difference between the cumulative number of BE of the right and left forefeet of all patients taken together (right forefoot = 167 BE versus left forefoot 157= BE, p=0.7). In addition, the mean number of BE considering the whole population was not significantly different when comparing the left and the right forefoot (left forefoot 8.72±4.68 versus right forefoot 9.28±4.28; p=0.59).

Number of BE at MTH: CT revealed a total of 258 BE at MTH (131 right sides versus 127 left sides; p=0.8). Comparing the mean number of BE present between right and left forefoot revealed a statistically significant difference only for the fifth MTH. Indeed, the right side was more affected (mean number of erosions 1.34±1.2 at the fifth right MTH versus 2.06±1.26 at the left fifth MTH; mean difference −0.67±1.19; p=0.029). No statistically significant difference was found for the other MTH (p>0.05).

Number of BE at PP: CT revealed a total of 66 BE at the base of the PP. There was no significant difference between the total number of BE on the right and the left forefeet (40 right forefeet versus 33 left forefeet, p=0.6). In addition, the mean number of BE at the PP was not statistically significant when comparing the corresponding PP of each side (p>0.05 for all values). On the contrary, when comparing the PP of the same side, the number of BE of the PP was significantly different only when comparing the first PP (0.73± 0.03) to the fifth PP of the right forefoot (mean difference= 0.02±0.07; p=0.0038).

Number of BE per quadrant: Table 1 details the BE number for each quadrant of each MTH and PP (first to fifth MTPJs) for the right and the left forefoot.

**Table 1:** Absolute number of BE per side for each quadrant of each MTH and PP. TOT: total.

Number of BE	Superior right	Fibular right	Plantar right	Tibial right	Superior left	Fibular left	Plantar left	Tibial left	Total
1° MTH	5	9	10	13	5	11	8	9	70
2° MTH	3	1	4	7	2	9	7	6	39
3° MTH	1	7	5	14	2	7	5	5	46
4° MTH	4	5	3	6	3	4	7	12	44
5° MTH	5	13	11	8	3	7	7	8	62
1° PP	1	3	4	9	0	3	2	2	24
2° PP	1	0	3	4	1	0	1	2	12
3° PP	0	1	1	2	0	1	1	1	7
4° PP	0	1	1	3	1	1	1	2	10
5° PP	0	2	2	3	1	2	0	4	14
Total	20	42	44	69	18	45	39	51	324

**Figure 3:**
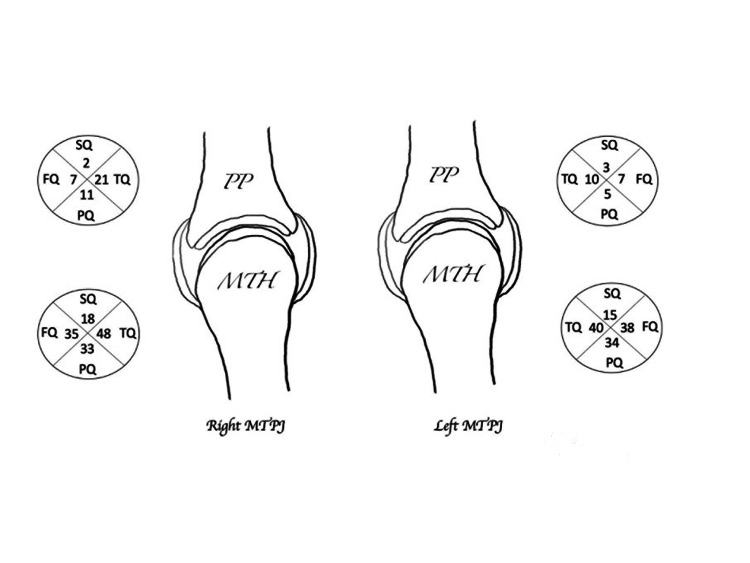
Cumulative number of bone erosions. In each quadrant for the MTH and the PP for the right and the left sides with the MTPJ from the first to the fifth MTPJ were taken together. PP: proximal phalanx, MTH: metatarsal head, SQ: superior quadrant, TQ: tibial quadrant (medial), PQ: plantar quadrant, FQ: fibular quadrant (lateral).

Figure 3 represents the cumulative number of BE in each quadrant of the MTH and PP (first to fifth MTPJs) for the right and the left forefoot.

Table 2 describes the cumulative number of BE, the percentage of BE in each quadrant for the MTH, the PP with the statistical values independently from the side, and the joint (MTPJ from first to fifth are taken together). On cumulative analysis, the superior quadrants were statistically less frequently affected than all other quadrants for both the MTH (p>0.0001) and the PP (p<0.001; Table 2). On the contrary, the tibial quadrant showed a statistically significantly higher number of erosions compared to all other quadrants for the MTH (p<0.03), the PP (p<0.01), and for the MTPJ considered as a whole (p<0.0001). The plantar and the fibular quadrants were equally affected (p<0.05), both having more BE compared to the superior quadrants and less BE compared to the tibial quadrants (Table 2).

**Table 2:** Cumulative number of bone erosions for the metatarsal heads. PP and MTPJ independently from the side and the joint (MTPJ from first to fifth are taken together). The p-value is provided for quadrants with a statistically significantly different number of BE than the null hypothesis of a random distribution (i.e., 25% of erosion for each of quadrants). MTPJ: metatarsophalangeal joint, BE: bone erosions, MTH: metatarsal head, PP: proximal phalange, SQ: superior quadrant, FQ: fibular quadrant, PQ: plantar quadrant; TQ: tibial quadrant, NS: no statistically significant difference than a random distribution of the bone erosion around the joint.

Quadrant	No. of BE MTH	(%)	No. of BE PP	(%)	No. of BE MTPJ (MTH+PP)	(%)
SQ	33	(12,8%); p>0.0001	5	(7.6%); p>0.001	38	(11.7%); p<0.0001
FQ	73	(28,3%); NS	13	(19.7%); NS	86	(26.5%);NS
PQ	67	(26%); NS	16	(24.3%); NS	83	(25.2%);NS
TQ	85	(32,9%); p>0.003	32	(48,4%); p>0.001	117	(36.1%); p>0.0001
Total no. of BE	258	(100%)	66	(100%)	324	(100%)

Topography of BE: Variance Component Analysis

The source of variance of the topography of BE was assessed considering the different levels of the analysis (Table 3).

**Table 3:** Variance components analysis. SD: standard deviation, CV: component of variance.

Variance component analysis	Variance components	SD	CV	CV (%)
Quadrant	0.133	0.365	1.62	75.6
Joint	0.036	0.190	0.844	20.5
Side	0	0	0	0.0
Patient	0.0069	0.083	0.369	3.9
Total	0.174	0.420	1.87	100

In the present study, a total of 324 positive quadrants for BE were identified in the 1140 quadrants; the proportion of BE is, therefore, 324/1440 = 0.225 (i.d. 22.5%). Therefore, being the percentage π = 0.225, the total variance is noted as σ², being σ² = π × (1 − π). Therefore, in the present table, the total variance (σ²) is calculated as follows:

σ² = 0.225 × (1 − 0.225) = 0.174

The main factor contributing to the overall variance was the quadrant (explaining 75.6% variance). The variance calculated for the different MTPJ (e.g., the first, the second, etc.) accounted for 20.5% of the variance. Variance depending on the patient accounted only for 3.9% of the total variance. Finally, no significant variance depended on the side.

## Discussion

The present study is the first to describe the location of BE at the MTPJ of both forefeet in patients with RA using a tomographic imaging technique, regardless of the predominantly symptomatic side. Previous studies assessing the location of BE at both forefeet were performed using radiographs and, thus, were limited by their bi-dimensional nature [16-18].

Unlike previous studies performed using MRI [10], we systematically mapped using isotropic CT images the BE topography at the MTFJ without focusing on the symptomatic but imaging both forefeet. Previously, only Boutry et al. [19] performed bilateral MRI in patients with RA, but they included the forefeet only in few selected cases, based on clinical symptoms.

In this study, we systematically assessed the topography of BE by dividing each joint surface into four quadrants, according to the scheme to map BE at the MCPJs used in previous studies using CT and HR-pQCT [8,15]. The analysis of 18 different patients resulted in the analysis of a total of 1440 quadrants (18 patient × 2 sides × 5 MTFJ joints × 2 joint surfaces × 4 quadrants for each joint surface = 1440 quadrants). This division of the joint surface allows counting the number of BE of distinct functional areas. At the MTFJ, the tibial and fibular aspects of the MTHs and PPs are subjected to the mechanical stress of the collateral ligaments at the enthesis. At the same time, the plantar areas of the joints are subject to mechanical stress due to body weight [8,10,19-22]. 

Our study provided some significant results. We observed that the absolute number of BE of a given forefoot is a poor predictor of the number of BE occurring on the contralateral side. This finding is significant because bilateral CT has been suggested as a reliable technique to quantify BE [12]. CT scoring for BE in RA was recently used, applying the OMERACT MRI scoring system to CT, focusing on the more symptomatic foot. However, a recent meta-analysis on the validity of the RA MRI score applied to the more symptomatic forefoot using the OMERACT filter concluded that even when using MRI, the OMERACT score is not related to all pathologic findings, including BE. Our study suggests that both forefeet should be assessed for a reliable evaluation of the BE.

Second, no difference in terms of the number of BE was found between the right and left sides. This result suggests that BE topography is not correlated to side dominance, largely favouring the right side in the general population. These results are in keeping with the results obtained by Yaku et al. [18], who studied the effect of handedness in joint involvement in patients with RA on radiographs. The authors assessed 28 different joints in 334 patients, including the MTPJ, and found no significant effect of handedness in determining the lateralization of the BE - including the MTPJ [18].

Third, there is a statistically significantly higher number of BE at the MTH compared to PP (258 vs 66). These data parallel previous MRI studies [10,19] reporting a higher number of BE at the MTH of the more symptomatic forefoot. Our results corroborate the hypothesis that the weight-bearing load on the MTH may induce a higher occurrence of BE compared to PP, given the higher mechanical demand.

Fourth, in our population, only the fifth MTH of both sides and the first proximal phalanx of the right side were more affected compared to their, respectively, paired joint surfaces. This finding is similar to results from previous studies using radiographs in which a higher rate of BE was observed at first and the fourth MTFJ [4] and at the fifth MTFJ [19,23-25].

Finally, BE was statistically more frequent at the tibial quadrant for both the MTH and PP in our population. The fibular and plantar quadrant were statistically more affected by BE than the superior quadrant. Our results agree with previous radiographic and sonographic investigations reporting the highest number of BE at the tibial aspects of the MTH [24,26,27].

However, these data disagree with the findings of Siddle et al. [10], reporting an absolute predominance of BE at the plantar aspect of the MTFJ. This discordance could be explained by the different definitions of the joint areas. In their study, the joint surface was divided into four areas by a vertical and horizontal line, making it difficult to attribute BE to the plantar or the lateral aspects of the joint. Even if most of the mechanical load is located at the plantar aspect of the MTFJ, stabilized by the plantar plate [28], our study suggests that the mechanical demand at the insertion of the collateral ligaments may favour the location of BE at the tibial and fibular aspect of the joints.

The biomechanical demand of the collateral ligaments at the MTPJ [18,28], along with that of the intermetatarsal “tie-bar” ligamentous system [18], is well established [28]. The higher rate of BE at the tibial aspect of the MTFJ lasts partially unexplained. Other factors such as the distribution of cortical micro-channels (CoMiCs) or plantar plate or capsule failure could determine BE's prevalent location at the tibial aspect of the MTFJ. Other studies using HR-pQCT for CoMiCs [15] or MRI to assess the integrity of the bone plate and collateral ligaments should be carried out to test these hypotheses.

The present study has several limitations. The population included in this study is limited to 18 patients. Our number of patients is like previous studies performed using CT or MRI on a single foot [10,19]. Multicentric studies should be performed to obtain a larger extent of data considering the high number of parameters considered in the statistical analysis. In addition, we did not consider in our analysis laboratory test results such as the C-reactive protein (CRP) level or the Disease Activity Score (DAS) used in clinical practice. However, previous studies showed no correlation between the overall disease activity, local clinical symptoms at MTPJ, and BE [10].

Furthermore, we did not consider as covariates the presence of factors favouring the onset of BE, including joint subluxation and bone plate failure, because these findings are difficult to detect or questionable on CT images performed in a non-weight-bearing supine position. Concomitant deformities of the foot [25] and capsular and plantar plate failure [10], modifying the area of higher mechanical demand at the forefeet were suggested as possible factors to determine the topography of BE in MTFJs in different populations of patients. Finally, only the topography of the largest BE for each joint surface was considered in the analysis. The topography of small size BE could have a different spatial pattern of anatomical location.

## Conclusions

The CT assessment of both forefeet showed that BE were more commonly found at the tibial aspect of the MTFJ, mostly at the MTH. The plantar and fibular aspect of the MTFJ had a lower rate of BE, with a similar incidence, suggesting that the biomechanical demand of the collateral ligaments at the MTPJ may play a key role in determining the topography of BE at the forefeet.
